# The Chronically Blocked Ear – A dive into external auditory exostosis

**DOI:** 10.1259/bjrcr.20220010

**Published:** 2022-03-31

**Authors:** Bianca Montebello, Stephanie Vella, Reuben Grech

**Affiliations:** 1 Department of Medical Imaging, Mater Dei Hospital, Msida, Malta

## Abstract

Numerous papers have reported the presence of reactive but benign bony exostosis in the external auditory canal of swimmers; of both cold and warm water. This outgrowth may lead to stenosis of the canal with associated complications such as repeated cerumen impaction, infections and also hearing loss. In this case report, we will present the case of a 62-year-old gentleman who was referred for imaging by an ENT specialist following difficulty with visualisation of the tympanic membrane during otoscopy.

## Clinical presentation and findings on imaging

A 62-year-old gentleman presented to ENT with a history of increased frequency of ear infections and impaction of cerumen. On otoscopic examination, it was noted that the patient had significant bilateral narrowing of the external auditory canal (EAC) which impaired visualisation of the tympanic membrane; more notable on the right than on the left. For better characterisation of this narrowing, the patient was referred for high-resolution CT (HRCT) of the temporal bones.

The patient also disclosed a history of troublesome removal of impacted cerumen; oftentimes requiring anaesthesia for this procedure in order to overcome the discomfort experienced. The patient has been aware of the presence of bony outgrowths for the past 30 years; he was previously offered surgical management which he declined. As his symptoms progressed to more frequent infections and accumulation of cerumen, he presented again to ENT.

When delving further into the patient’s history; he recalled being an avid-free diver on a daily basis during the summer months. This took place during his early teens to late adulthood but over the past 15 years, he decreased his participation in this aquatic activity. Whilst diving, the patient did not make use of any protective equipment such as swimming caps and ear plugs. The patient denied diving during the winter months.

**Figure 1.**
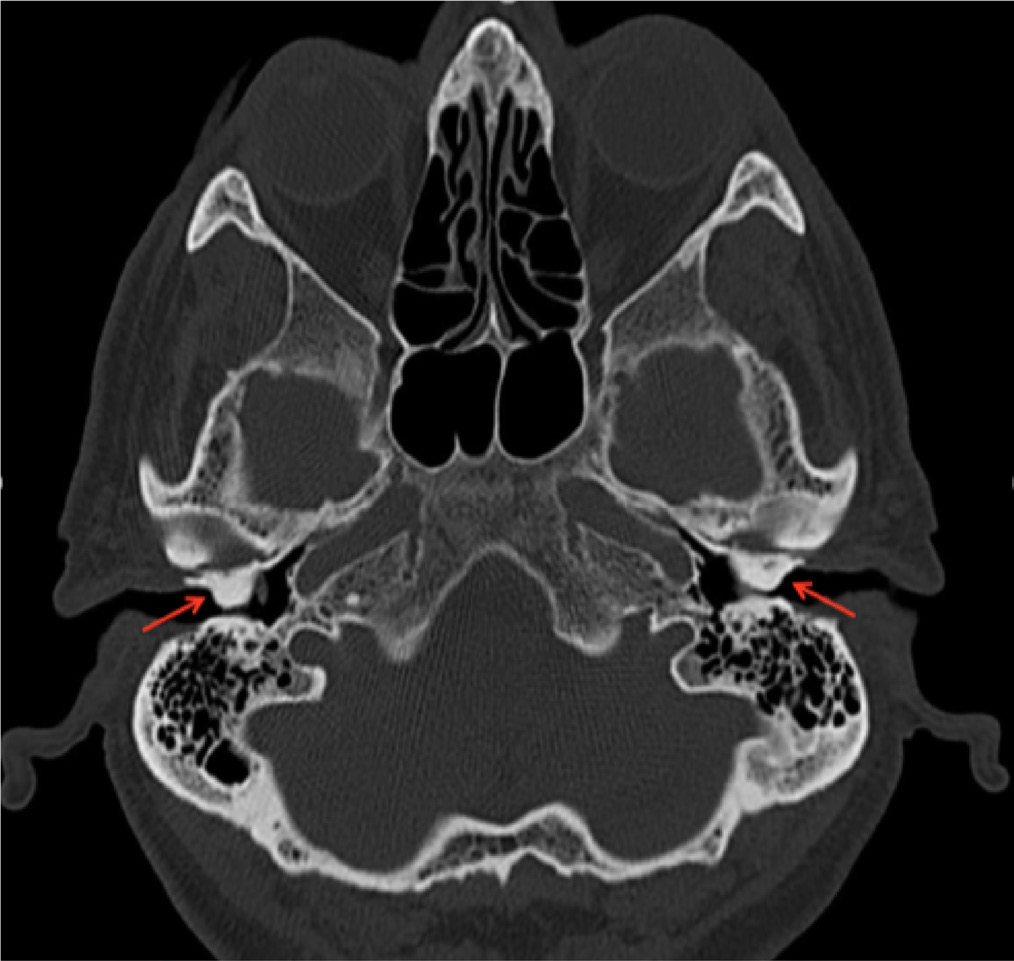
Axial high-resolution CT of the temporal bone demonstrating bilateral broad-based focal circumferential bony overgrowths (arrow) of the right and left osseous external auditory canal. These are causing significant narrowing of the lumen, with a residual luminal diameter of 1–2 mm bilaterally.

**Figure 2.**
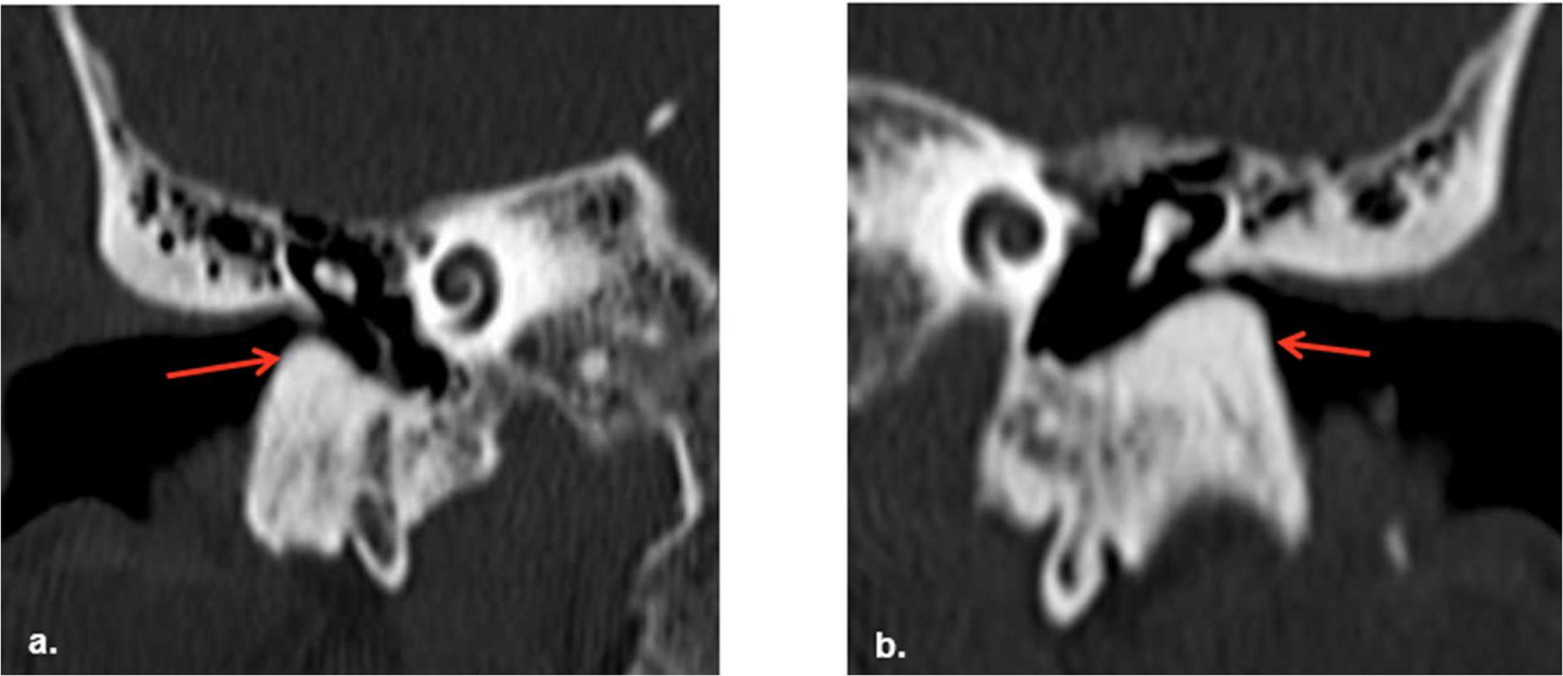
Coronal reformats again demonstrating bilateral osseous outgrowths (arrows) within the external auditory canal (a. Right and b. Left)

On HRCT, the patient was noted to have bilateral dense and sessile outgrowths extending from the osseous part of the EAC. These outgrowths are noted to be causing occlusion of the canal on axial and coronal imaging. No soft tissue densities are visualised within the canals or bony erosions. These changes are visualised in Figure 1 (axial section) and Figure 2 (coronal section a and b).

## Discussion

External auditory exostosis (EAE) are benign and irreversible growths which are visualised in the EAC during otoscopy and radiological imaging. They have been dated back to the era of the prehistoric man and these were useful identifiers for cultures where there was frequent aquatic exposure.^1^ The hyperplasia is a potential result of chronic thermal exposure such as cold-water immersion or inflammatory processes such as recurrent otitis externa.^2^

The pathophysiology behind these benign growths is uncertain. Landefeld et al postulated that EAE arose secondary to irritation of the mucoperiosteum present in the external auditory meatus which stimulated osteoblastic activity. This increase in osteoblast activity was then followed by fibrosis and ossification which ultimately lead to new bone growth. It is a process which was believed to be a protective mechanism against cold water and cold air.^1^ Simas et al found that the prevalence of EAE in warm-water and cold-water surfers was similar.^3^ However, the current literature favours the theory that cold-water exposure, especially that colder than 19℃, and length of exposure leads to new bone formation.^4^

Patient presentation will vary depending on the severity of the exostosis present. They would typically present with complaints which occur secondary to the exostosis such as conductive hearing loss, fullness within the ear and otalgia. These symptoms may be the result of cerumen impaction, otitis externa or else rupture of the tympanic membrane.^1^ Prior to the use of CT, the diagnosis and grading of exostosis relied on the patient symptomatology and otoscopic examination.^5^ The otoscopic grading system ranges from 0 (No identifiable exostosis) to 3 (>67% obstruction) and will often determine the approach required for patient management. However, as clinical examination can underestimate the degree of exostosis, gold-standard for diagnosis is imaging with CT.^5^ This is also of importance for planning if surgical treatment is being considered.^1^

On CT, these benign outgrowths are characterised by a dense protuberance on the osseous part of the EAC.^6^ They are typically seen bilaterally, located medial to the isthmus and would have a sessile broad base.^4^ This benign condition is rarely studied on imaging modalities.

Management will vary depending on the grade of severity. Prevention is the initial step in deterring the development and progression of this condition. A study carried out by Lambert et al delved into the correlation between ear protection and the development of exostosis. It concluded that the use of ear plugs had no correlation with EAE development. On the other hand, they found a correlation between the development of EAE and lack of usage of ear plugs.^7^ The mainstay of treatment is symptomatic relief; however, should the patient be highly symptomatic or else Grade 3, then surgical excision is advised.^5^

A patient presenting with a history of chronic seawater exposure along with visualisation of bilateral bony outgrowths in the EAC, majority of the cases will have a definitive diagnosis for EAE.

## Differential diagnosis

Conditions which could potentially produce similar symptomatology and imaging on CT include external ear osteoma and cholesteatoma. The most common pitfall differential would be an osteoma.

On petrous bone CT, the osteoma would present as a unilateral, pedunculated bony lesion which is lateral to the isthmus, unlike exostoses which are found medial. These osteomas are typically incidental findings.^4^

Other differential diagnoses also include keratosis obturans and medial canal fibrosis, both of which are close differentials for cholesteatomas. Table 1 offers a comprehensive list of features which would permit differentiation between potential diagnoses.^8^

**Table 1.** Differential diagnosis of radiological significance to exostosis^4,8^

	Exostosis	Osteoma	Cholesteatoma	Keratosis obturans	Medial canal fibrosis
**Location**	Bilateral	Unilateral	Unilateral	May be bilateral	Variable to the insult.
**Tissue associated with occlusion**	Broad-rased protuberance of the bone in the external canal; usually multiple	Pedunculated and typically solitary	Soft tissue with occasional presence of bony fragments within.^a^	Soft tissue plugs without bony fragments	Fibrotic tissue
**Bony involvement**	No erosion	No erosion	Periostitis and erosion of the underlying bone	No erosion	No erosion
**EAC calibre**	Narrowing of the EAC	Narrowing of the EAC	No change in EAC width	Widening of the EAC	No widening; Shallow ‘Pseudofundus’

EAC, external auditory canal.

a The absence of bony components within the soft tissue does not exclude cholesteatoma as a differential.^8^

## Learning points

1. In its early stages, this condition may be overlooked on imaging due to the presence of minimal outgrowth.
2. The patient’s clinical history plays an important role in the contextualisation of imaging. Salient symptoms and signs include otalgia, bilateral conductive hearing loss and recurrent otitis media. There may also be visualisation of the exostosis during otoscopy.
3. CT plays an important role in the diagnosis, grading of severity and surgical planning.
4. Appreciation of bilateral dense and sessile protuberances with a background history of aquatic activity in cold water is in keeping with a diagnosis of EAE.
5. Awareness of alternative conditions affecting the EAC are vital.

## References

[1] Landefeld K, Bart RM, Lau H . Surfer's Ear. [Updated 2021 Aug 1]. In: StatPearls [Internet]. Treasure Island (FL): StatPearls Publishing; 2021.

[2] Patwari S, Joshi S, Chadaga H . Bilateral exostosis of the external auditory canal: Surfer’s ear. 2021. 10.4103/0028-3886.241360 30233044

[3] Simas V, Hing W, Rathbone E, Pope R, Climstein M . Auditory exostosis in Australian warm water surfers: a cross-sectional study. BMC Sports Sci Med Rehabil 2021; 13: 52. doi: 10.1186/s13102-021-00281-5 33990216 PMC8122542

[4] Kumar T, Pulickal G . Imaging of External Ear Malformations, Canal Stenosis and Exostosis. Temporal Bone Imaging Made Easy 2021; 71–75. doi: 10.1007/978-3-030-70635-7_11

[5] Climstein M, Simas V, DeBeliso M, Walsh J . A novel method for the determination of exostosis severity in the external auditory canal. Clin Otolaryngol 2021; 46: 1247–50. doi: 10.1111/coa.13824 34142441

[6] Brea B, Roldán Fidalgo A . Imaging Diagnosis of Benign Lesions of the External Auditory Canal. Acta Otorrinolaringologica (English Edition) 2013; 64: 6–11. 10.1016/j.otoeng.2013.02.008 22901893

[7] Lambert C, Marin S, Esvan M, Godey B . Impact of ear protection on occurrence of exostosis in surfers: an observational prospective study of 242 ears. Eur Arch Otorhinolaryngol 2021; 278: 4775–81. doi: 10.1007/s00405-021-06609-8 33555441

[8] Pulickal G, Tan T, Chawla A , eds. Temporal Bone Imaging Made Easy. Switzerland: Springer; 2021., pp.66–75. doi: 10.1007/978-3-030-70635-7

